# Patient-Generated Subjective Global Assessment and Phase Angle as Predictors of Survival in Cancer Patients

**DOI:** 10.7759/cureus.88972

**Published:** 2025-07-29

**Authors:** Marco Aurélio Ribeiro, Fernanda Peria, Gleici da Silva Perdona, Anderson Marliere Navarro

**Affiliations:** 1 Division of Nutrition and Metabolism, Department of Internal Medicine, School of Medicine of Ribeirão Preto, University of São Paulo, Ribeirão Preto, BRA; 2 Division of Oncology, Department of Medical Images, Hematology and Clinical Oncology, School of Medicine of Ribeirão Preto, University of São Paulo, Ribeirão Preto, BRA; 3 Division of Epidemiology and Biostatistics, Department of Social Medicine, School of Medicine of Ribeirão Preto, University of São Paulo, Ribeirão Preto, BRA

**Keywords:** bioelectrical impedance analysis, cancer, cancer survival, mortality, nutritional assessment, nutritional support, oncology malnutrition, pg-sga, phase angle

## Abstract

Background: Malnutrition is a common and serious complication among cancer patients that negatively affects treatment response, quality of life, and overall survival. Tools such as the Patient-Generated Subjective Global Assessment (PG-SGA©) and phase angle (PA), which is derived from bioelectrical impedance analysis (BIA), have shown promise in identifying nutritional risk. Their effectiveness as mortality predictors requires further validation.

Objective: Investigate the association between different nutritional assessment approaches, including the PG-SGA, body mass index (BMI), fat-free mass index (FFMI), and phase angle, and overall survival in patients undergoing systemic cancer treatment.

Methods: A prospective study was conducted with 111 adult patients treated at a tertiary university hospital. Nutritional assessments were performed using the PG-SGA and BIA, and clinical and oncological data were collected. Survival analysis was performed using Kaplan-Meier curves, and mortality-associated variables were identified using Cox proportional hazards regression models.

Results: During the follow-up period, 51.4% of patients died. PG-SGA classification was strongly associated with mortality; severely malnourished patients had a 4.68-fold higher risk of death than well-nourished patients (hazard ratio [HR]: 4.68; p < 0.001), even after adjusting for tumor type. FFMI also emerged as a relevant marker, with higher mortality observed in patients with low lean body mass. Phase angle showed a significant association with survival in univariate analysis, though this association lost significance after adjusting for clinical stage.

Conclusion: These findings suggest that a comprehensive nutritional assessment that integrates clinical and bioelectrical parameters may provide valuable information for risk stratification in oncology. The PG-SGA classification stood out as an independent predictor of mortality, and phase angle demonstrated potential as a complementary marker. The results underscore the importance of early nutritional screening as an essential component of multidisciplinary cancer care.

## Introduction

Malignant neoplasms are among the most prevalent causes of morbidity and premature mortality worldwide, constituting a substantial public health concern. According to the most recent GLOBOCAN estimates, published in 2022, approximately 19.3 million new cancer cases and 10 million cancer-related deaths were documented in 2020. These figures reflect the growing impact of cancer on aging and urbanized populations across the globe [1-3].

In Brazil, the National Cancer Institute (INCA) has projected approximately 704,000 new cancer cases per year for the 2023-2025 triennium, with 483,000 cases when non-melanoma skin cancers are excluded. The most prevalent tumors include those of the female breast, prostate, and gastrointestinal tract, whose treatments often result in significant nutritional complications [4].

Malnutrition is a common occurrence among patients receiving oncological treatment. This phenomenon is the result of numerous factors, including those related to the tumor itself, the adverse effects of treatment, and the metabolic changes associated with cancer. This condition is closely associated with reduced treatment response, impaired functional capacity, increased clinical complications, and lower overall survival. Recent studies have demonstrated that early nutritional intervention has a substantial impact on the quality of life and clinical outcomes of these patients [5-7].

According to the updated guidelines of the European Society for Clinical Nutrition and Metabolism (ESPEN, 2022), it is recommended that all cancer patients undergo nutritional screening to allow for the early identification of risk and the subsequent implementation of timely interventions. Personalized nutrition has emerged as a prominent strategy to mitigate treatment-related complications and enhance adherence [7].

Among the available methods for nutritional assessment, the Patient-Generated Subjective Global Assessment (PG-SGA©) is distinguished by its extensive acceptance and its efficacy in detecting malnutrition. This instrument systematically evaluates weight loss history, dietary intake changes, functional capacity, nutrition impact symptoms (NIS), and anthropometric alterations, providing a clear classification of patients as well-nourished, moderately malnourished, or with suspected or severe malnutrition. Furthermore, PG-SGA scores have demonstrated a correlation with significant clinical outcomes, including postoperative complications, reduced chemotherapy tolerance, and survival [8-11].

Another widely used method for nutritional assessment in oncology is bioelectrical impedance analysis (BIA), particularly phase angle (PA), which is derived from the resistance and reactance of electrical current through body tissues [12,13]. PA has been demonstrated to serve as a reliable indicator of cellular membrane integrity and body hydration status. It is associated with body composition and prognosis in a variety of clinical conditions. In cancer patients, low PA values have been associated with decreased survival, diminished quality of life, and a heightened prevalence of sarcopenia [14,15].

Recent studies underscore the significance of integrated tools such as PG-SGA and PA in clinical practice for a comprehensive nutritional assessment of oncology patients. However, there are still gaps in the literature regarding the association between these nutritional markers and survival in heterogeneous cancer populations undergoing systemic treatment. These discrepancies underscore the necessity for studies exploring the combined application of such tools to guide nutritional interventions and optimize clinical outcomes [7].

The objective of this study was to investigate the association between different nutritional assessment methods, including the PG-SGA, body mass index (BMI), fat-free mass index (FFMI) and PA obtained by BIA, and overall survival in cancer patients undergoing systemic treatment. Patients with different cancer types were included to ensure a heterogeneous and clinically representative oncology population, and the analysis was adjusted for tumor type since biological differences may influence nutritional status, treatment response and clinical outcomes. The primary outcome was overall survival, assessed using Kaplan-Meier curves, and mortality-associated variables were identified using Cox proportional hazards regression models.

## Materials and methods

This prospective observational study was conducted at the oncology treatment unit of a Brazilian public university hospital recognized as a referral center for managing complex cancer cases. The research protocol was reviewed and endorsed by the local Research Ethics Committee (protocol no. 712449), thereby ensuring adherence to the established ethical standards for human subject research. All participants provided written informed consent, thereby confirming their understanding of the study's objectives and procedures.

Patient population

The study spanned 30 months, with patient recruitment initiated in the second half of 2014 and follow-up completed in March 2017. This time frame enabled the consecutive enrollment of a clinically representative sample of oncology patients receiving antineoplastic therapy, providing a sufficient observation period for survival analysis. A total of 111 adult patients (age ≥ 21 years) were consecutively enrolled from the oncology outpatient population based on predefined eligibility criteria.

The inclusion criteria encompassed patients undergoing systemic treatment for various types of cancer, irrespective of histological classification, clinical stage, or therapeutic category. Patients were required to be receiving systemic cancer treatment, as the primary aim of the study was to evaluate the association between nutritional status and survival in individuals already under antineoplastic therapy. This methodological criterion ensured that the findings reflected the population actively undergoing treatment, avoiding the inclusion of newly diagnosed patients or those without active therapy, whose prognosis and nutritional status could differ substantially. This comprehensive inclusion strategy was developed to enhance the representativeness of the heterogeneous oncology population under follow-up at the oncology department. The duration of follow-up varied depending on the date of the initial assessment, with a median of 16 months (interquartile range: 7.0-23.5 months).

Patients were excluded from the study if they had received treatment for less than one month, to avoid early-survival bias. Patients with cardiac pacemakers and other electronic implantable devices (e.g., implantable cardioverter defibrillators) were excluded as a safety precaution, following manufacturers’ recommendations, due to the theoretical risk of interference with the low-intensity electrical current used in BIA. This criterion was adopted to ensure ethical rigor. Additionally, conditions that significantly alter body conductivity (such as severe edema or amputations) may also interfere with BIA accuracy and should be considered in future studies.

Oncological data

Information regarding patients' oncological profiles was obtained from their electronic medical records and supplemented by clinical assessments performed by experienced oncologists. Tumor staging was determined according to the Union for International Cancer Control (UICC) TNM classification, 8th edition, which is based on the characteristics of the primary tumor, involvement of regional lymph nodes, and the presence of metastases at diagnosis. Patients were classified into stages I, II, III, and IV, with stages III and IV designated as advanced disease according to standardized staging criteria. Treatment regimens were consistent with the most recent guidelines from the National Comprehensive Cancer Network (NCCN, 2024) [16]. These protocols underscore the personalization of systemic therapy based on tumor type, molecular biomarkers, and patients' clinical conditions, ensuring standardization and comparability across the analyzed cases.

Nutritional assessment

Nutritional assessment was conducted by trained evaluators, specifically clinical dietitians with experience in oncology and certified in the use of the PG-SGA and anthropometric measurements. Inter-rater reliability was ensured through standardized training sessions and periodic supervision by a senior oncology dietitian. Assessments were performed while patients waited for chemotherapy infusion. Body weight and height were measured using calibrated electronic scales and wall-mounted stadiometers, following the guidelines established by the World Health Organization (WHO) and the Pan American Health Organization (PAHO). Measurements were performed with patients wearing light clothing, without shoes, and scheduled before chemotherapy infusion to minimize treatment-related fluid shifts. Specific cutoff points were established for adults and older adults (age ≥65 years).

Patients were requested to document their body weight from one to six months before the preliminary evaluation, and this information was corroborated through the consultation of their medical records. When discrepancies were identified between self-reported weight and medical records, data from medical records were prioritized. These data were used to calculate the retrospective BMI at six months before the assessment, allowing comparative analysis with nutritional status at the time of evaluation.

The nutritional assessment instrument employed was the PG-SGA, a tool widely recognized for its sensitivity (60-99%) and specificity (70-86%) in identifying malnutrition among cancer patients [17,18]. The questionnaire includes items addressing recent weight loss, changes in dietary intake (quantity and consistency), functional limitations, and NIS that could impede nutritional intake. It also evaluates metabolic stress associated with malignancy and physical examination findings such as fluid retention (e.g., edema, ascites) and loss of muscle or fat stores. The full categorical classification was used to classify patients into three categories: A (well-nourished), B (moderately malnourished or at nutritional risk), and C (severely malnourished).

Bioelectrical impedance analysis

The assessment of body composition was conducted using bioelectrical impedance analysis (BIA), a method of measuring body composition through electrical resistance. This assessment was facilitated by the Quantum BIA 101 Q RJL System, a device manufactured in Clinton Township, Michigan, USA. Measurements were conducted in the morning or early afternoon, with patients fasting for at least four hours, after bladder emptying, avoiding strenuous physical activity for 12 hours and resting in a supine position for 10 minutes prior to assessment to ensure standardized conditions. This non-invasive method enables precise estimation of fat-free mass (FFM) and fat mass (FM), expressed both in absolute values (kilograms) and as a percentage of total body weight [19]. The FFMI was calculated by dividing FFM by height squared. The diagnostic cutoffs for sarcopenia were established at less than 15 kg/m² for women and less than 17 kg/m² for men [3]. Furthermore, PA, calculated using resistance (R) and reactance (Xc), was used as a marker of cell membrane integrity and hydration status. Reactance reflects cell membrane capacitance and body cell mass. Patients were classified as having low or normal PA based on age- and sex-specific reference values derived from Barbosa-Silva et al. (2005) [20]. Table 1 summarizes the adapted reference values used in this study.

**Table 1:** Reference Phase Angle Values by Age Group and Sex Source: Adapted and restructured from Barbosa-Silva et al., 2005 [20]. Values represent approximate summaries of reported means.

Age Group (years)	Mean Phase Angle ± SD (Men)	Mean Phase Angle ± SD (Women)
			18–29	7.96 ± 0.61	6.92 ± 0.88
30–49	7.89 ± 0.85	6.73 ± 0.86
50–69	7.14 ± 1.00	5.81 ± 0.89
≥70	6.19 ± 0.97	7.04 ± 0.85

Mortality data

During the subsequent follow-up period, 57 patients (51.4%) succumbed to their conditions. The initial step in the process was outcome identification, which was performed by consulting electronic health records. In instances where recent updates were not available, patient attendance at the oncology service was reviewed. For subjects who had discontinued follow-up, telephone contact was initiated with family members to confirm the death and record the exact date. This enabled the calculation of the time interval between the nutritional assessment and the outcome.

Data analysis

For descriptive analysis, the mean, median, and standard deviation were calculated for the quantitative variables. Absolute frequencies and percentages were reported for categorical variables. The association between PG-SGA classification and qualitative variables was assessed using Fisher's exact test [21]. For quantitative variables, Student's t-test and ANOVA were applied, or the corresponding non-parametric tests, including the Wilcoxon-Mann-Whitney test [21] and the Kruskal-Wallis test [21], when assumptions for parametric tests were not satisfied.

The survival curves for the various PG-SGA classifications were generated using the Kaplan-Meier method, and comparisons between these curves were performed using the Log-Rank test. To this end, two separate Cox proportional hazards regression models were constructed to evaluate the association between overall survival and the study's primary nutritional predictors: PA and the PG-SGA. The decision to utilize separate models was based on the distinct nature of these variables. Phase angle is an objective bioelectrical measure, whereas PG-SGA is a subjective tool based on clinical and nutritional findings.

Although this study focused on the categorical classification of PG-SGA (A, B, or C), we acknowledge the potential prognostic value of the total PG-SGA score as a continuous variable and intend to explore this approach in future analyses. All analyses were conducted using R software, with a significance level set at 5%.

## Results

At the time of nutritional assessment, most patients were non-elderly (n = 79, 71.2%), female (n = 73, 65.8%), and had advanced tumor staging, with stage III in 27 patients (24.3%) and stage IV in 62 patients (55.9%), as shown in Table 2. The most prevalent tumor types were breast cancer (n = 31, 27.9%) and gastrointestinal tract cancer (n = 42, 37.8%), reflecting the pattern observed in populations with similar epidemiological characteristics. A significant proportion of the patients received palliative treatment (n = 66, 59.5%), highlighting the clinical complexity of this population and the importance of multidisciplinary support in its management.

**Table 2:** General Characteristics of 111 Patients Undergoing Systemic Cancer Treatment # Cases with no staging available correspond to hematological malignancies.

Variable	Mean (SD)	
Age (years)	56.2 ± 14.8	
Non-elderly: Elderly (%)	71.2, 28.8	
	Percentage (%)	
Female: Male (%)		65.8, 34.2
Tumor staging (%)		
No staging available #	6.3	
I	1.8	
II	11.7	
III	24.3	
IV	55.9	
Primary tumor site (%)		
Skin	1.8	
Head and neck	3.6	
Breast	27.9	
Digestive tract	37.8	
Lung	7.2	
Gynecological	7.2	
Hematological and lymphoid	8.1	
Urological	6.3	
Treatment intent (%)		
Definitive	4.5	
Neoadjuvant / Adjuvant	36	
Palliative	59.5	
Treatment modality (%)		
Chemotherapy	85.6	
Chemotherapy plus Radiotherapy	14.4	

Nutritional classification

Table 3 presents a comparison of BMI classifications from six months before the assessment with those obtained at the time of the study. The proportion of patients classified as obese decreased significantly (from 33.3% to 17.1%; p = 0.01), while the frequency of underweight individuals increased significantly (from 8.1% to 20.7%; p = 0.001). These results suggest a progressive impact of disease and treatment on body composition, emphasizing the importance of continuous nutritional monitoring.

**Table 3:** BMI Classification in 111 Patients Undergoing Systemic Cancer Treatment p-value obtained using the McNemar test.

BMI classification	Six months before		At assessment	Test statistic; p-value
Obesity (n, %)		37 (33.3)	19 (17.1)	χ²(1) =18.00; p<0.001
Eutrophic / Overweight (n, %)		65 (58.6)	69 (62.2)	
Underweight (n, %)		9 (8.1)	23 (20.7)	

Classification by PG-SGA, FFMI, and phase angle

As illustrated in Table 4, the distribution of patients is depicted according to the PG-SGA, the FFMI, and the PA. According to the PG-SGA classification system, many patients (n = 76, 68.5%) were classified as well-nourished, while 25.2% (n = 28) were identified as malnourished based on their FFMI. Concerning PA, 30.6% of patients (n = 34) exhibited values below the established cutoffs, suggesting potential impairment of cellular membrane integrity and compromised nutritional status.

**Table 4:** Classification According to Patient-Generated Subjective Global Assessment (PG-SGA), Fat-Free Mass Index (FFMI), and Phase Angle (PA)

PG-SGA (%)	
Well-nourished (A)	68.5
Moderately malnourished (B)	20.7
Severely malnourished (C)	10.8
FFMI (%)	
Adequate	74.8
Malnutrition	25.2
PA (%)	
Non-low	69.4
Low	30.6

Correlation between nutritional variables

Correlation analysis, summarized in the matrix presented in Table 5, revealed statistically significant associations between BMI and PG-SGA classification (p = 0.012) and between BMI and FFMI (p < 0.001). Furthermore, the PG-SGA exhibited significant correlations with the FFMI (p = 0.023) and low phase angle (p = 0.048), indicating consistency among the various nutritional assessment methods. However, no statistically significant correlations were observed between phase angle and either BMI (p = 0.124) or FFMI (p = 0.461). This suggests that phase angle may reflect distinct components of nutritional status.

**Table 5:** Correlation Between Nutritional Variables (BMI, Patient-Generated Subjective Global Assessment (PG-SGA), Fat-Free Mass Index (FFMI), and Low Phase Angle (PA)) ρ: Spearman’s rank correlation coefficient, S: test statistic from Spearman’s correlation; p-values are two‐tailed.

Variable	BMI	PG-SGA	FFMI	Low PA
BMI	1.000	-0.237 (S= 281848.43; p=0.012)	0.802 (S=45033.80; p=<0.001)	-0.147 (S=261354.89; p=0.124)
PG-SGA	-0.237 (S=281848.43; p=0.012)	1.000	-0.215 (S=276926.40; p=0.023)	0.188 (S=185000.22; p=0.048)
FFMI	0.802 (S=45033.80; p=<0.001)	-0.215 (S=276926.40; p=0.023)	1.000	-0.071 (S=244046.56; p=0.461)
Low PA	-0.147 (S=261354.89; p=0.124)	0.188 (S=185000.22; p=0.048)	-0.071 (S=244046.56; p=0.461)	1.000

Association between FFMI, PG-SGA, and mortality

The analysis examined the relationship between malnutrition, as determined by the PG-SGA, and lean tissue depletion, as estimated by FFMI, as well as their correlation with mortality during the follow-up period. As shown in Table 6, patients with low FFMI exhibited a significantly higher mortality rate (75.0%) than those with adequate FFMI (43.4%).

**Table 6:** Association between fat-free mass index (FFMI), Patient-Generated Subjective Global Assessment (PG-SGA), and mortality during follow-up (n = 111) Chi-square test was used for comparisons involving FFMI, and Fisher’s exact test was applied to PG-SGA categories due to small cell frequencies.

Variable	Death n (%)	No Death n (%)	Total n (%)	Test statistic; p-value
Adequate FFMI	36 (43.4)	47 (56.6)	83 (74.8)	χ²(1)=8.38; p=0.004
Low FFMI	21 (75.0)	7 (25.0)	28 (25.2)	
PG-SGA A	34 (44.7)	42 (55.3)	76 (68.5)	χ²(2)=6.49; p=0.039
PG-SGA B	13 (56.5)	10 (43.5)	23 (20.7)	
PG-SGA C	10 (83.3)	2 (16.7)	12 (10.8)	

Furthermore, patients categorized as severely malnourished (PG-SGA Category C) had a notably higher mortality rate (83.3%) than those in Categories A and B, and a statistically significant association was found between PG-SGA categories and mortality (Fisher's exact test, p = 0.039). Additionally, a higher prevalence of tumors impairing oral intake was observed among Category C patients (p = 0.0122). These findings reinforce the clinical relevance of both FFMI and PG-SGA as prognostic indicators in oncological nutritional assessment.

Impact of nutritional status on clinical outcomes in oncology patients

This study reveals meaningful associations between patients’ nutritional status and clinical outcomes, particularly survival. A variety of statistical tests were applied according to the nature and distribution of the data (Table 7).

**Table 7:** Descriptive Analysis According to Patient-Generated Subjective Global Assessment (PG-SGA) Classification p-values were calculated using the Kruskal–Wallis test for Age; Chi-square test for Gender, Tumor staging, Treatment modality, and BMI classification; and Fisher’s exact test (p-values only, no test statistic reported) for fat-free mass index (FFMI) classification and mortality.

Variable	PG-SGA A (n=76)	PG-SGA B (n=23)	PG-SGA C (n=12)	Test statistic; p-value
Age (years)	55.9 ± 14.7	54.0 ± 16.2	62.3 ± 11.9	χ²(2)=1.42; p=0.493
Gender (%)				
Female	54 (71.1)	15 (65.2)	5 (41.7)	χ²(2)=4.05; p=0.132
Male	22 (28.9)	8 (34.8)	7 (58.3)	
Tumor Staging (%)				
Not recorded	6 (7.9)	1 (4.3)	0 (0.0)	χ²(6)=9.22; p=0.162
I	2 (2.6)	0 (0.0)	0 (0.0)	
II	12 (15.8)	1 (4.3)	0 (0.0)	
III	20 (26.3)	3 (13.0)	4 (33.3)	
IV	36 (47.4)	18 (78.3)	8 (66.7)	
Treatment Modality (%)				
Definitive	4 (5.3)	1 (4.3)	0 (0.0)	χ²(4)=3.75; p=0.442
Neoadjuvant / Adjuvant	31 (40.8)	5 (21.7)	4 (33.3)	
Palliative	41 (53.9)	17 (73.9)	8 (66.7)	
BMI Classification (%)				
Underweight	11 (14.5)	6 (26.1)	6 (50.0)	χ²(4)=10.04; p=0.040
Eutrophic / Overweight	49 (64.5)	14 (60.9)	6 (50.0)	
Obesity	16 (21.1)	3 (13.0)	0 (0.0)	
FFMI Classification (%)				
Adequate	61 (80.3)	19 (82.6)	3 (25.0)	p=0.000
Low	15 (19.7)	4 (17.4)	9 (75.0)	
Death (%)				
No	42 (55.3)	10 (43.5)	2 (16.7)	p=0.037
Yes	34 (44.7)	13 (56.5)	10 (83.3)	

The chi-square test confirmed that patients with low FFMI had significantly higher mortality rates than those with an adequate FFMI (p = 0.000), which reinforces the prognostic relevance of FFMI in oncology. Due to low expected frequencies in some categories, Fisher's exact test was used for comparisons involving PG-SGA classifications. The results showed that patients in PG-SGA Category C (severely malnourished) had significantly higher mortality rates (83.3%) and a higher incidence of tumors in advanced anatomical sites (p = 0.037).

The Kruskal-Wallis test was applied to compare age across PG-SGA categories due to the non-normal distribution of age. Although patients in PG-SGA Category C tended to be older (mean age = 62.3 years), this difference was not statistically significant (p = 0.493).

Additional descriptive analyses revealed worse clinical and nutritional parameters in severely malnourished patients, including a higher prevalence of underweight BMI (50.0%), low FFMI (75.0%), and palliative treatment (66.7%). Although tumor staging and gender did not reach statistical significance, a clear gradient was observed, with category C patients presenting more frequently with stage IV disease and being predominantly male.

Overall, the use of tailored statistical approaches enabled clinically relevant patterns to be identified, even within subgroups of limited size. These findings emphasize the value of routine nutritional assessments in cancer care, not merely for supportive purposes, but as an essential element of prognostic evaluation.

Survival analysis and relative risk of death according to PG-SGA staging: Kaplan-Meier curves and Cox regression model

**Figure 1:**
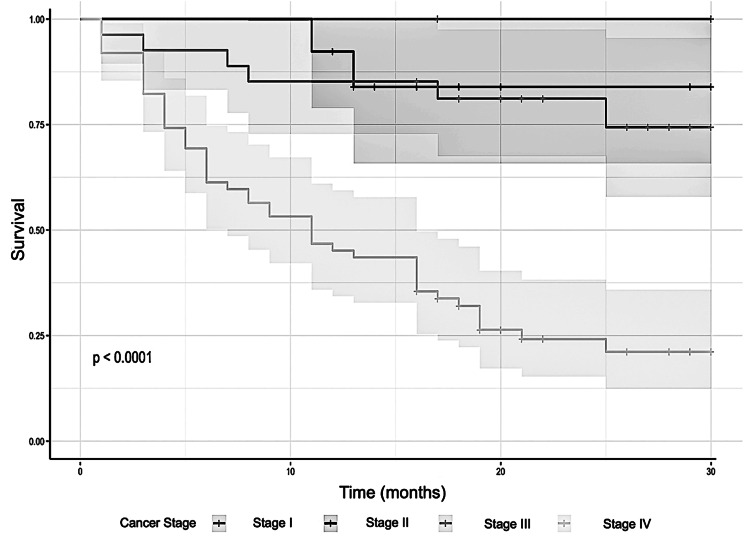
Kaplan-Meier Curves for Overall Survival According to Cancer Stage The following study will examine Kaplan-Meier survival curves for overall survival in oncology patients stratified by nutritional classification using the Patient-Generated Subjective Global Assessment (PG-SGA). The curves demonstrate a marked decrease in survival among patients classified as severely malnourished (C) compared to those classified as B and A. This difference was confirmed to be statistically significant by the log-rank test (X2(3)=29.69, with p < 0.001).

Figure 1 shows the Kaplan-Meier survival curves, which are divided according to nutritional status as determined by the PG-SGA. The log-rank test revealed statistically significant differences in survival between the nutritional categories (χ²(3) = 29.69; p < 0.001), which confirms the prognostic value of the PG-SGA in oncology settings.

Patients in Category C (severely malnourished) had a significantly lower survival probability than those in Categories A and B, and the steep decline in their survival curve highlights the high mortality risk associated with severe malnutrition. Conversely, patients categorized as well-nourished (Category A) demonstrated the most favorable survival trajectory throughout the follow-up period.

To quantify the risk further, a Cox proportional hazards model was applied, adjusting for potential confounding variables. This revealed that patients in PG-SGA Category C had a hazard ratio (HR) of 3.82, indicating a 3.82-fold higher risk of mortality over time compared to patients in Category A, and this association remained significant after controlling for other clinical factors. This suggests that malnutrition independently predicts worse survival outcomes.

Integrating Kaplan-Meier analysis and Cox regression provides a comprehensive evaluation of survival trends and a robust statistical estimate of mortality risk. These findings emphasize the clinical usefulness of PG-SGA as not only a nutritional screening tool, but also a valuable prognostic indicator that can inform therapeutic decisions and care prioritization in oncology, particularly for individuals categorized as severely malnourished.

Table 8 presents the results of the Cox proportional hazards regression model, adjusted for nutritional status (PG-SGA classification) and tumor type, to evaluate their association with mortality risk among oncology patients.

**Table 8:** Results of the Cox Regression Model Adjusted for Patient-Generated Subjective Global Assessment (PG-SGA) Classification and Tumor Type as Covariates for Mortality in Oncology Patients Cox proportional‐hazards regression model for mortality in oncology patients, adjusted for PG-SGA classification and tumor type. PG-SGA A was set as the reference category. Coefficient (β) is the estimated log‐hazard, Hazard Ratio (HR) = exp(β), and p-values are from the Wald test.

PG-SGA Classification	Coefficient (β)	Hazard Ratio (HR)	Wald z	p-value
A	-	-		-
B	0.39	1.48	1.06	0.289
C	1.54	4.68	3.28	0.001

Of the 111 patients assessed, 76 (68.5%) were classified as PG-SGA A (well nourished), 23 (20.7%) as PG-SGA B (moderately malnourished), and 12 (10.8%) as PG-SGA C (severely malnourished). The regression model used PG-SGA A as the reference category. Patients in PG-SGA Class B demonstrated a non-significant increase in mortality risk compared to the reference group, with a hazard ratio (HR) of 1.48 (β = 0.39; p = 0.289). Although this result was suggestive of a trend towards a worse prognosis, it did not reach statistical significance.

**Figure 2:**
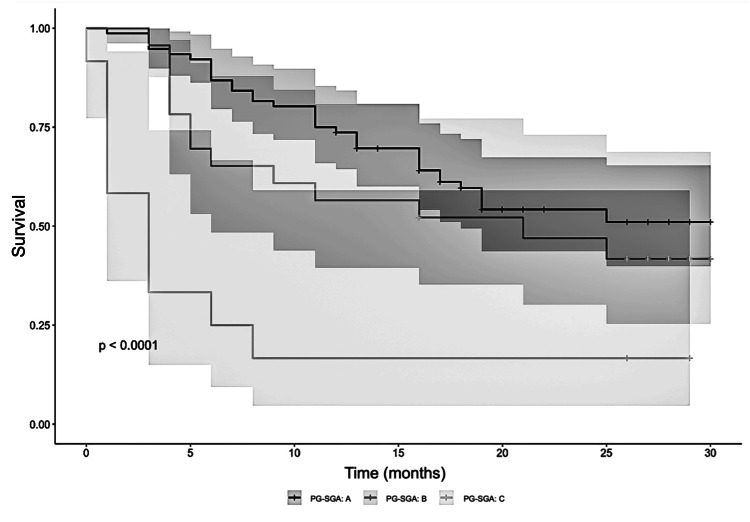
Adjusted Overall Survival in Oncology Patients Stratified by Patient-Generated Subjective Global Assessment (PG-SGA) Classification The following investigation sought to determine the impact of nutritional stratification by PG-SGA on the adjusted cumulative survival of oncology patients, utilizing a Cox regression analysis. Patients in Class C exhibited a 4.68-fold elevated risk of mortality compared to individuals in Class A, a finding that demonstrated robust statistical significance (p < 0.001). These results underscore the independent prognostic value of the PG-SGA.

However, patients classified as PG-SGA C exhibited a statistically significant and markedly elevated risk of death, with an HR of 4.68 (β = 1.54; p = 0.001) (Figure 2). This implies that, after adjusting for tumor type, the probability of death over time was more than four times greater in severely malnourished patients than in their well-nourished counterparts.

These results highlight the importance of nutritional status as an independent prognostic factor in oncology. While moderate malnutrition (PG-SGA B) may not significantly affect survival on its own, severe malnutrition (PG-SGA C) poses a substantial mortality risk. These findings advocate the inclusion of comprehensive nutritional assessments and interventions as standard components of oncological care, particularly for patients at high nutritional risk.

Phase angle analysis

Table 9 shows the distribution of clinical and nutritional variables according to phase angle classification, grouping patients as either low or non-low phase angle. The results reveal significant correlations between low phase angle values and more severe clinical conditions at the time of nutritional assessment.

**Table 9:** Distribution of Variables According to Low and Non-low Phase Angle p-values were calculated using Student’s t-test for age; chi-square for categorical variables (gender, tumor staging, chemotherapy modality, BMI classification), and the Fisher’s exact test for fat-free mass index (FFMI) classification, and death outcome.

		Phase Angle		
		No (n=77)	Yes (n=34)	Test statistic; p-value
Age	(Mean ± SD)	57.10 (14.90)	54.26 (14.72)	t(64)=0.93; p=0.354
Gender (%)	Female	53 (68.8)	21 (61.8)	χ²(1)=0.26; p=0.610
	Male	24 (31.2)	13 (38.2)	
Tumor Stage at assessment (%)	Not recorded*	3 (3.9)	4 (11.8)	χ²(3)=7.99; p=0.046
	I	2 (2.6)	0 (0.0)	
	II	12 (15.6)	1 (2.9)	
	III	22 (28.6)	5 (14.7)	
	IV	38 (49.4)	24 (70.6)	
Chemotherapy Modality (%)	Definitive	1 (1.3)	4 (11.8)	χ²(2)=11.36; p=0.003
	Neoadjuvant/Adjuvant	34 (44.2)	6 (17.6)	
	Palliative	42 (54.5)	24 (70.6)	
Grouped BMI Classification at Assessment (%)	Underweight	12 (15.6)	11 (32.4)	χ²(2)=4.37; p=0.112
	Eutrophic/Overweight	52 (67.5)	17 (50.0)	
	Obesity	13 (16.9)	6 (17.6)	
FFMI Classification (%)	Adequate	61 (79.2)	22 (64.7)	p=0.154
	Malnourished	16 (20.8)	12 (35.3)	
Death (%)	No	42 (54.5)	12 (35.3)	p=0.068
	Yes	35 (45.5)	22 (64.7)	

Patients with a low phase angle were significantly more likely to have an advanced tumor stage (stage IV) (70.6% vs. 49.4%; χ²(3) = 7.99; p = 0.046) and to be receiving palliative care (70.6% vs. 54.5%; χ²(2) = 11.36; p = 0.003). These findings support the interpretation of phase angle as a potential marker of disease severity in oncology settings. However, no statistically significant differences were observed between the groups regarding age, gender, BMI classification, or FFMI, although there was a numerical trend towards a higher frequency of underweight and malnourished individuals in the low phase angle group. FFMI results assessed using Fisher's exact test suggest that, although body composition is often linked to phase angle, this association did not reach significance in the current sample.

Furthermore, mortality was higher among patients with a low phase angle (64.7%) than among those with a non-low phase angle (45.5%), with a borderline p-value of 0.068. While not statistically significant, this finding suggests a potential trend towards poorer outcomes in this subgroup and warrants further investigation with larger sample sizes.

These results suggest that low phase angle values are associated with markers of clinical deterioration, particularly tumor burden and treatment intent, even if they are not strongly linked to traditional nutritional metrics such as BMI or FFMI in this sample. These findings strengthen the case for incorporating phase angle into routine nutritional and prognostic evaluations in oncology care.

Table 10 summarizes the results of a Cox proportional hazards regression model examining the association between low phase angle, tumor stage, and mortality in oncology patients. The reference categories were patients with a normal phase angle and those in tumor stages I/II.

**Table 10:** Cox Regression Model Results With Low Phase Angle and Tumor Restaging as Covariates Cox proportional‐hazards regression model for mortality, adjusted for Low Phase Angle and clinical restaging. Reference categories are Low Phase Angle = No and Tumor Restaging = Stages I/II. Coefficient (β) is the estimated log‐hazard, Hazard Ratio (HR) = exp(β), and p‐values are from the Wald test.

Variable	Coefficient (β)	Hazard Ratio (HR)	Wald z	p-value
Low Phase Angle: Yes	0.42	1.52	1.48	0.139
Tumor Stage: III	0.40	1.49	0.49	0.624
Tumor Stage: IV	2.04	7.68	2.80	0.005

Patients with low phase angle values were 1.53 times more likely to die than those with normal phase angle values (hazard ratio (HR) = 1.52; β = 0.42), although this difference was not statistically significant (p = 0.139). The observed effect size suggests a possible contribution of phase angle to mortality risk, even if it is not independently predictive within this model.

Tumor stage emerged as a key prognostic factor. Patients with stage IV tumors showed a significantly increased risk of death, with a hazard ratio of 7.68 (β = 2.04; p = 0.005), indicating a markedly higher likelihood of mortality compared to those in the early stages. For stage III, the hazard ratio was 1.49 (β = 0.40), but this result was not statistically significant (p = 0.624).

These findings suggest the strong prognostic influence of advanced tumor burden, while also suggesting that phase angle may offer additional prognostic insight, especially when interpreted alongside other clinical variables. Including phase angle in routine assessments could improve risk stratification, particularly in settings where disease severity is multifactorial.

**Figure 3:**
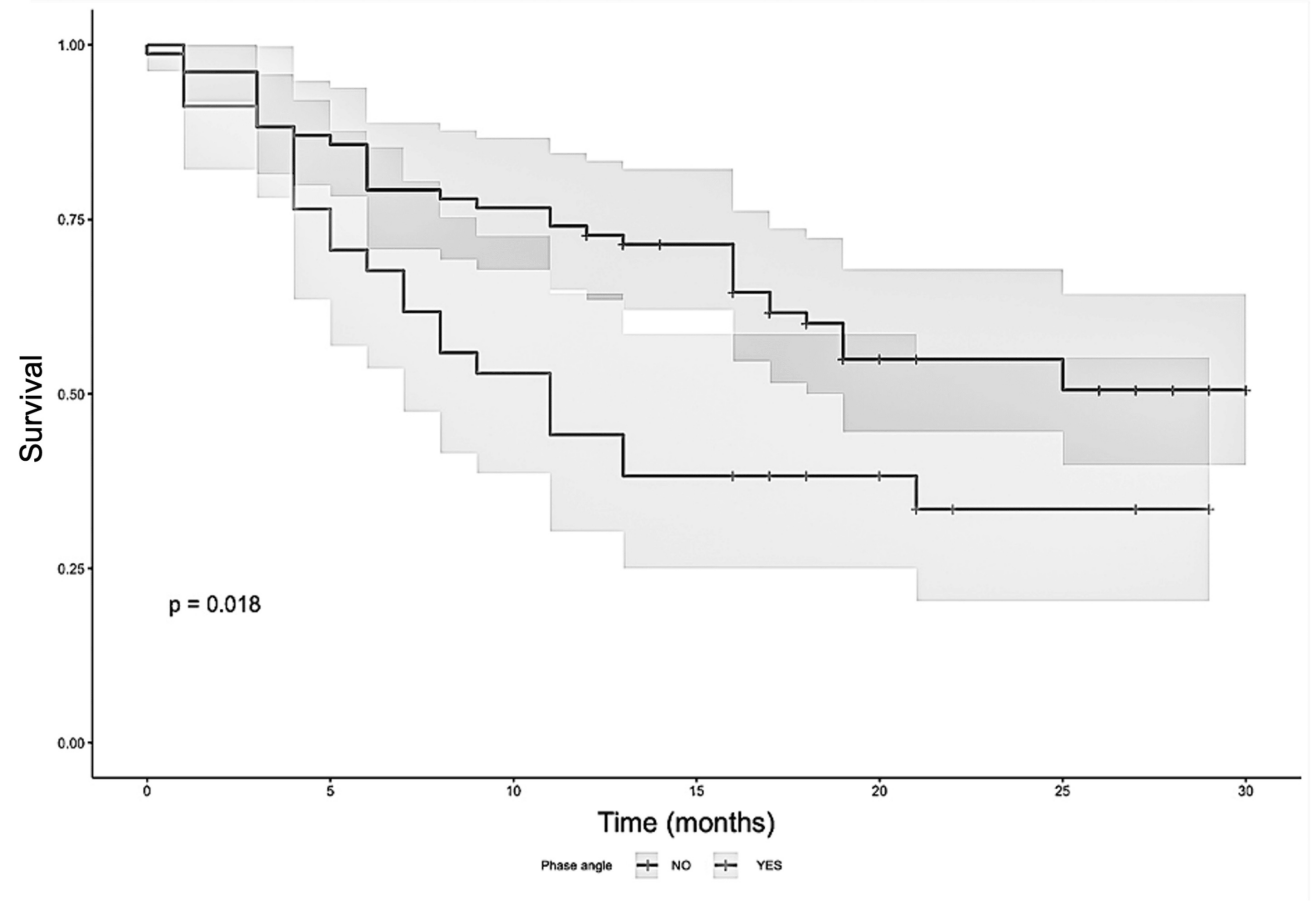
Kaplan-Meier Survival Curves Stratified by Phase Angle Classification Kaplan-Meier curves for overall survival were generated to compare patients with phase angle (PA) values classified as low versus within reference ranges according to sex and age. Patients exhibiting a physician-assessed PA below the standard reference range demonstrated a 12-month survival rate of 44%, which is significantly lower than the 73% survival rate observed among patients with a PA within the reference range. The observed discrepancy was found to be statistically significant based on the log-rank test (X2(1)=5.57; p = 0.018), thereby indicating a probable prognostic value for PA.

As Figure 3 shows, the Kaplan-Meier survival curves reveal a statistically significant difference in overall survival between patients with a low phase angle and those with values within the reference range. Patients with a low phase angle had substantially lower survival probabilities, with a 12-month survival rate of 44% compared to 73% for patients with normal phase angle values. This difference was confirmed by the log-rank test (X2(1)=5.57; p = 0.018). While these findings suggest an association between phase angle and survival outcomes, other clinical variables, such as tumor stage and treatment modality, appear to exert a stronger prognostic influence.

## Discussion

The results of the present study underscore the importance of comprehensive nutritional assessment in oncology patients, integrating both objective and subjective methods that allow risk stratification and the prediction of adverse clinical outcomes. The PG-SGA has been demonstrated to be a reliable instrument for identifying patients at high nutritional risk, with classification in category "C" being associated with a significantly increased risk of mortality. These findings are consistent with those of previous studies that have associated severe malnutrition in oncology with increased protein catabolism, reduced functional capacity, and worsened clinical outcomes, including overall survival [8-10,22,23].

Moreover, the data on PA, obtained by BIA, further substantiates its utility as an independent prognostic marker, consistent with prior studies [24-26]. Patients exhibiting low PA values demonstrated diminished survival, thereby providing consistent evidence that PA reflects early changes in cell membrane integrity and metabolic status, which are critical aspects of the nutritional condition in oncology patients [12,20,27-30]. The significance of PA lies in its ability to capture dimensions of nutritional status that are not detected by traditional anthropometric parameters, such as BMI or body composition indices [15,28]. This finding aligns with previous research indicating that PA is more closely associated with clinical prognosis than with isolated body composition metrics [15,29,30].

Another salient aspect that emerged from the analysis was the observed correlation between malnutrition and tumor staging, predominantly in stages III and IV. Patients with more advanced disease demonstrated a higher prevalence of malnutrition, reflecting the cumulative impact of tumor-driven catabolism and systemic treatment toxicity on nutritional status. A body of research has previously documented that heightened systemic inflammation, a hallmark of advanced malignancies, serves to exacerbate lean body mass loss and metabolic dysfunction [5,7,18].

The progressive decline in obesity and the concomitant increase in underweight cases throughout the follow-up period are indicative of substantial changes in body composition, underscoring the necessity for early and ongoing nutritional intervention. Nutritional management must be considered an integral component of multidisciplinary oncology care, especially in patients undergoing palliative treatment, where the objective is to preserve quality of life and reduce clinical complications [7,20,21].

The findings from the Kaplan-Meier and Cox regression analyses substantiate that patients categorized as severely malnourished (Class C) by PG-SGA are confronted with a 4.68-fold elevated risk of mortality in comparison to well-nourished patients (Class A). This lends support to the notion that PG-SGA is a sensitive tool with the capacity to predict adverse clinical outcomes and guide targeted nutritional interventions [5,9].

Despite the absence of substantial correlations between PA and other nutritional markers, such as BMI and FFMI, the robust correlation with survival indicates that PA may serve as an early indicator of physiological changes that could potentially lead to overt malnutrition. This finding underscores the significance of PA as an autonomous and supplementary prognostic indicator in the nutritional evaluation of oncology patients [10,12,15]. Although the statistical significance of PA was reduced after adjustment for clinical stage, this should be interpreted with caution, as low PA values are frequently observed in patients with advanced disease, reflecting the interaction between nutritional status and disease severity [27-29]. Therefore, this reduction does not imply a lower clinical relevance of PA but suggests that part of its prognostic value may be mediated by disease severity. PA thus remains an independent and complementary indicator to PG-SGA for nutritional risk stratification and prognosis in oncology [12,13,24,26].

This study lends further credence to the notion that nutritional assessment constitutes a critical element in the clinical management of cancer patients. Malnutrition, present in up to 80% of oncology patients in advanced stages, is directly associated with reduced functional capacity, increased complications, and higher mortality [5,7]. In this context, the integration of objective and subjective methods, such as PA and PG-SGA, signifies a promising approach to the early identification of patients at high nutritional risk and to the guidance of specific and individualized nutritional interventions. As demonstrated in previous studies, the PG-SGA scale exhibits a high degree of sensitivity in detecting malnutrition [12,20,22,23].

The implementation of these methodologies in clinical practice is of pertinence in vulnerable populations, such as patients in advanced disease stages or under palliative care, where the primary objectives are the maintenance of quality of life and the prevention of nutritional complications [7]. The extant literature also emphasizes that nutrition-based interventions grounded in comprehensive assessments can reduce treatment-related complications, improve therapeutic response, and potentially extend overall survival [5,9]. Consequently, the systematic implementation of these methodologies within the framework of routine oncology care has been demonstrated to enhance the efficacy of management and to facilitate personalized care, thereby aligning with international guidelines on nutritional support in oncology [7].

Limitations

While this study is valuable in contributing to understanding the link between nutritional parameters and survival in cancer patients, its limitations should be acknowledged. The sample size, though adequate for the analyses, may have constrained the detection of smaller associations, particularly for phase angle, which lost significance after adjusting for clinical stage. However, the sample's clinical representativeness was maintained by including patients with various tumor locations and disease stages. The diversity, detailed in the methodology and summarized in Table 2, enhances the applicability of the PG-SGA and phase angle across different oncological settings.

The study was conducted in a single tertiary university hospital in Brazil, which may limit the generalizability of the findings to other healthcare settings. However, the inclusion of patients with various tumor types, clinical stages, and therapeutic categories aimed to enhance the representativeness of the heterogeneous oncology population.

A notable methodological limitation is the absence of international standardization for phase angle cut-offs in cancer populations, making direct comparisons with other studies difficult. Nonetheless, the reference values used are from well-established literature, supporting consistent interpretation. Future multicenter studies and efforts to standardize PA cutoffs in oncology are warranted.

Retrospective body weight data was partly based on patient self-report, which could introduce recall bias. To mitigate this, checks using medical records were performed and future studies should strengthen this approach to further reduce bias. The analysis also categorized PG-SGA scores (A, B, or C), while future research should investigate the prognostic potential of its continuous score.

Regarding follow-up, 30 months was considered sufficient for assessing overall survival, the study's main endpoint. This does not constitute a methodological limitation. Still, longer follow-up periods in future research could help clarify long-term outcomes and the overall impact on nutrition.

## Conclusions

This study demonstrated that the combined use of the PG-SGA and phase angle provides relevant prognostic information in oncology patients. Malnutrition identified through PG-SGA and low phase angle values was associated with higher mortality, particularly in advanced-stage cancer. Although phase angle showed reduced statistical significance after adjustment for clinical stage, it remains a clinically relevant marker of cellular integrity and nutritional risk, complementing the PG-SGA, which was confirmed as an independent predictor of mortality.

These findings reinforce the importance of integrating nutritional assessment into routine oncology care, highlighting its role in risk stratification and clinical decision-making. Future studies should include larger, multicenter cohorts and explore additional biomarkers, as well as the prognostic value of the continuous PG-SGA score, to optimize nutritional interventions in cancer care.
